# Quality of dying after acute stroke

**DOI:** 10.1177/23969873211041843

**Published:** 2021-09-05

**Authors:** Hendrik Reinink, Marjolein Geurts, Constance Melis-Riemens, Annemarie Hollander, Jaap Kappelle, Bart van der Worp

**Affiliations:** 1 Department of Neurology and Neurosurgery, 526115 University Medical Center Utrecht, Utrecht, The Netherlands; 2 Department of Neurology 6993 Erasmus Medical Center, Rotterdam, The Netherlands

**Keywords:** Acute stroke, palliative care, end-of-life care

## Abstract

**Introduction:**

There is a lack of evidence concerning the palliative needs of patients with acute stroke during end-of-life care. We interviewed relatives of patients who deceased in our stroke unit about the quality of dying and compared their experiences with those of nurses.

**Patients and Methods:**

Relatives of 59 patients were interviewed approximately 6 weeks after the patient had died. The primary outcome was a score assessing the overall quality of dying on a scale ranging from 0 to 10, with 0 representing the worst quality and 10 the best quality. We investigated the frequency and appreciation of specific aspects of the dying phase with an adapted version of the Quality of Death and Dying Questionnaire. The nurse who was most frequently involved in the end-of-life care of the patient completed a similar questionnaire.

**Results:**

Family members were generally satisfied with the quality of dying (median overall score 8; interquartile range, 6–9) as well as with the care provided by nurses (9; 8–10) and doctors (8; 7–9). Breathing difficulties were frequently reported (by 46% of the relatives), but pain was not. Unsatisfactory experiences were related to feeding (69% unsatisfactory), inability to say goodbye to loved ones (51%), appearing not to have control (47%), and not retaining a sense of dignity (41%). Two-thirds of the relatives reported that palliative medication adequately resolved discomfort. There was a good correlation between the experiences of relatives and nurses.

**Discussion and Conclusion:**

Most relatives were satisfied with the overall quality of dying. Negative experiences concerned feeding problems, not being able to say goodbye to loved ones, sense of self control and dignity, and breathing difficulties. Experiences of nurses may be a reasonable and practical option when evaluating the quality of dying in acute stroke patients.

## Introduction

Approximately half of stroke-related deaths occur in hospital,^1^ and palliative and end-of-life care are therefore common practice at a stroke unit.^1,2,3^ Research on end-of-life care has mainly focused on cancer patients, whereas patients dying as a consequence of stroke may have different palliative needs.^4,5^ An American Heart Association statement concluded that there is a striking lack of evidence concerning optimal palliative care practices in patients with acute stroke,^1^ including a large knowledge gap regarding specific needs of dying stroke patients and their families.^2,6^ Pain, agitation and dyspnoea have been reported as the main symptoms in dying stroke patients,^1^ but studies that systematically analysed symptoms in these patients are scarce and are mostly based on review of symptoms reported in hospital charts.^7^ Impairments in communication with the patient are a frequent complicating factor in the interpretation of symptoms of dying stroke patients.^2,6^ Healthcare professionals therefore often include the experience of family members when assessing if palliative needs have been met.

In the current study, we aimed to assess the experience of family members about symptoms during the terminal phase and the ‘quality of dying’ in patients who deceased in our stroke unit. We also ascertained the opinion of nurses about the quality of dying in the same patients.

## Methods

We prospectively included consecutive patients with acute ischaemic stroke, intracerebral haemorrhage (ICH) or subarachnoid haemorrhage (SAH) from October 2017 to June 2020 who died after a decision to withdraw life-sustaining measures and to start end-of-life care on the stroke unit of the University Medical Center Utrecht, a tertiary referral centre in the Netherlands. In this stroke unit, patients in whom life-sustaining measures are withdrawn are being cared for in a single room. Palliative care is primarily provided by nurses, residents in neurology and supervising neurologists. A palliative care consultative service is available upon request. Patients in whom no decision to start palliative care was made before death were excluded from this study.

The physician who had overseen the end-of-life care sent the first contact person of the deceased patient a letter of condolence approximately four weeks after the patient had died. This letter included an introduction of the study and a do-not-contact-me return form. Subsequently, relatives who did not return the do-not-contact-me form were contacted by telephone by one of two trained research nurses who asked permission to be interviewed at home approximately two weeks later. Informed consent was obtained from all relatives for the use of the information they provided during the interview. During the COVID-19 outbreak, interviews were performed by telephone. The Medical Ethics Committee of our hospital waived approval of the study under the Dutch Medical Research Involving Human Subjects Act.

The questionnaires that were used during the interviews can be found in the Supplementary Material. The research nurse started the interview by asking relatives the question: ‘Overall, how would you rate the quality of dying of your family member on a scale ranging from 0 to 10, with 0 representing the worst quality and 10 the best?’ In addition, relatives were asked to rate the care provided by nurses, the care provided by doctors, the quality of communication and support provided by healthcare professionals, as well as to assess the letter of condolence and the interview on the same 0–10 scale. Relatives were also asked to score the length of the end-of-life phase after the decision to stop all curative treatments on the same scale, with 0 representing that the process was ‘much too short’ and 10 representing that it was ‘much too long’. Subsequently, the research nurse and relatives completed the ICU version of the Quality of Death and Dying Questionnaire (QODD-ICU).^8^ The QODD is a validated questionnaire developed for palliative research that may be used for interviewing bereaved significant others after death.^9^ The QODD-ICU consists of 25 of the original 31 questions that evaluate symptoms, experiences and perceptions about the dying process in the last week of life.^10^ Each item of the QODD instrument consists of two parts: a frequency component and a quality component. The first assesses the frequency of a particular symptom or experience. Depending on the item, family members are asked to indicate frequencies across a range from 0 (none of the time) to 5 (all of the time) or dichotomously with yes (event/experience occurred) or no (event/experience did not occur). In the second part, the family members are asked how the particular symptom or experience affected the quality of dying on a 0–10 scale: 0 indicates a terrible experience and 10 an almost perfect experience.^8^ Family members can skip questions if they feel that they cannot rate the experience or if an item was not applicable.

To better reflect the situation of our population, we disregarded QODD-ICU items on mechanical ventilation, dialysis and healthcare costs. We added questions about whether the patient was short of breath and whether palliative medication successfully relieved signs of discomfort. Additionally, the nurse who had cared most for the patient in the end-of-life phase was asked to fill out the adapted QODD-ICU for nurses within 7 days after the death of the patient (Supplementary Material).^11^

We collected information from the medical records about patient characteristics (age, sex, pre-stroke functional dependency (modified Rankin Scale)); stroke characteristics (dates and times of onset and hospital admission, stroke type, stroke severity (National Institute of Health Stroke Scale)) and details about end-of-life care (date and time of start of end-of-life care, location where this was started (stroke unit, emergency room or ICU), use of opioids or benzodiazepines, moment of death). The start of the end-of-life phase was defined as the moment the treating physician had made a note in the patient record that life-sustaining measures were withdrawn and that the goal of further care was aimed at optimizing patient comfort.

The primary outcome was the relatives’ experience of the quality of dying, defined as the score given in response to the first overall question. Secondary outcomes were (1) the nurses’ opinion on the quality of dying, defined as their score on the first overall question; (2) the frequency component of each item of the QODD-ICU scored by either relatives or nurses; and (3) the quality rating component of individual items of the QODD-ICU scored by either relatives or nurses. For descriptive analysis of symptoms of discomfort, we dichotomized the frequency component of each item of the QODD-ICU to ‘never or sometimes’ (score 0–2) and ‘most or all of the time (score 3–5)’ and dichotomized the quality rating component to ‘unsatisfactory’ (score 0–5) and ‘satisfactory’ (score 6–10), in line with the general grading system in Dutch schools, where scores of six or higher are considered satisfactory. We disregarded a QODD-ICU item if the quality component was scored by less than half of the relatives, as we considered these items not to be representative for our population. In contrast to previous studies, we therefore decided not to use the QODD-ICU total (average) score as an outcome in our study, which is calculated by adding up the scores of the quality rating components of the individual items and dividing this by the number of items answered.^8^ All scores are displayed as median with corresponding IQR. All percentages are displayed as proportion of the number of valid responses to each question.

Differences in scores between relatives and nurses was analysed with the Wilcoxon signed-rank test. If relatives declined to participate or could not be contacted, only the nurses’ scores were used. To assess potential selection bias, we compared patient characteristics between participating and non-participating relatives. In addition, we compared the nurses’ score on the summary question between patients with a relative who participated and those without using the Mann–Whitney U test.

To analyse the relation of patient and clinical characteristics on the primary outcome, we used a linear regression model. The following independent variables were used for univariate analysis: age, sex, stroke type (ischemic stroke, ICH or SAH), length of the end-of-life phase (in hours), time between hospital admission and withdrawal of life-sustaining measures (in hours), use of opioids (yes/no), use of benzodiazepines (yes/no) and the level of consciousness at start of palliative phase (assessed with the Glasgow Coma Scale). We hypothesized that symptoms related to uncomfortable breathing would be the most frequently reported sign of discomfort and would be the main target for palliative medication. Therefore, we performed an additional regression analysis with the same independent variables and the score on the quality rating component of the QODD-ICU item about breathing comfort as the dependent variable. Variables were included in multivariate models if *p* < 0.10 in the univariate analysis.

We used Pearson correlation coefficient to analyse the relationship between the length of the end-of-life phase (in hours) and the relatives’ opinion of the duration of the end-of-life phase (score 0–10).

The criterion for statistical significance was set at α = 0.05. No adjustments were made for multiplicity of testing, as we considered the analyses hypothesis-generating.

## Results

**Figure 1.**
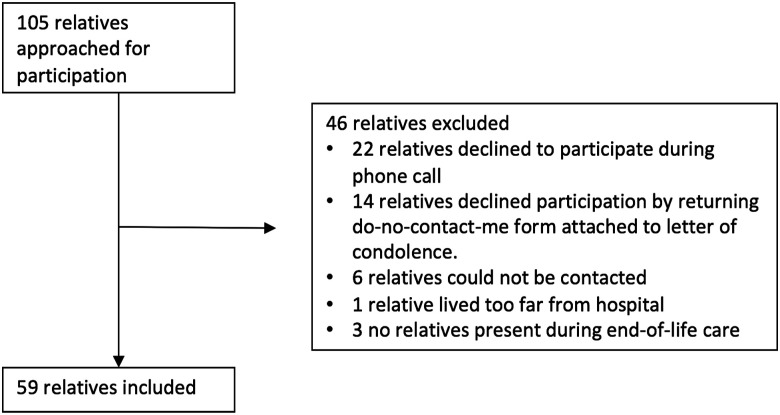
Flowchart of included relatives and reasons for non-participation.

During the study period, 105 patients died on the stroke unit after a decision to withdraw life-sustaining measures and to start end-of-life care. Relatives of 59 patients agreed to be interviewed. Relatives of 36 patients declined participation and six relatives could not be contacted (Figure 1). Palliative care consultants were involved in the care of six of the 59 patients (10%). Baseline characteristics were balanced for patients whose relatives were interviewed and those who were not (Table 1). In addition, the nurses’ overall scores for quality of dying were not different for participating and non-participating relatives (*p* = 0.56).

**Table 1.** Baseline characteristics

	All patients (*n* = 105)	Relatives interviewed (*n* = 59)	Relatives not interviewed (*n* = 46)	*p*-value
Age (mean + SD)	75.8 (12.0)	77.0 (12.3)	74.3 (11.6)	0.24
Male sex (%)	48 (46%)	24 (41%)	24 (52%)	0.33
Type of stroke (%)				0.95
Ischemic stroke	44 (42%)	24 (41%)	20 (44%)	
ICH	44 (42%)	25 (42%)	19 (41%)	
SCH	17 (16%)	10 (17%)	7 (15%)	
Pre-stroke mRS (%)				0.26
0	23 (22%)	14 (24%)	9 (20%)	
1	11 (11%)	4 (7%)	7 (15%)	
2	19 (18%)	11 (19%)	8 (17%)	
3	19 (18%)	11 (19%)	8 (17%)	
4	11 (11%)	9 (15%)	2 (4%)	
Unknown	22 (21%)	10 (17%)	12 (26%)	
Length EOL-phase (hours; median + IQR)	24.7 (6.0–45.6)	19.5 (5.5–48.4)	31.4 (6.4–44.4)	0.46
Location start of EOL-care				0.56
Stroke unit	55 (52%)	30 (51%)	25 (54%)	
Intensive care unit	22 (21%)	11 (19%)	11 (24%)	
Emergency department	28 (27%)	18 (31%)	10 (22%)	
NIHSS at admission (median + IQR)	21 (16–34)	22 (17–37)	21 (13–29)	0.49
GCS at admission (median + IQR)	9 (5–13)	9 (4–11)	10 (6–13)	0.27
Latest GCS before EOL-care (median + IQR)	6 (4–9)	6 (4–9)	7 (5–8)	0.66
Use of opioids used (%)	78 (75%)	45 (76%)	33 (73%)	0.91
Use of benzodiazepines (%)	30 (29%)	15 (25%)	15 (33%)	0.56

EOL indicates end-of-life; mRS: modified Rankin Scale; NIHSS: National Institutes of Health Stroke Scale: Glasgow Coma Scale; ICH: intracerebral haemorrhage; SAH: subarachnoid haemorrhage.

Six QODD-ICU items could not be included in the analysis because they were completed by less than half of the relatives. The deleted QODD items included questions about whether the patient felt at peace with dying; laughed and smiled; cleared up bad feelings with others; had a spiritual ceremony before death; had discussed preferences for end-of-life care or had funeral arrangements in order.

### Experiences of relatives

**Figure 2.**
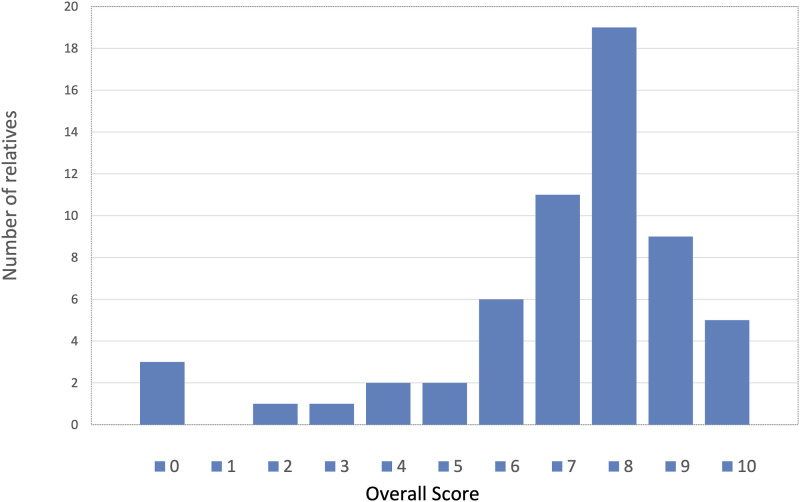
Distribution of relatives’ overall experience of the quality of dying.

Overall, the relatives were satisfied with end-of-life care (median overall score 8; IQR, 6–8), with 50 relatives (85%) scoring the overall experience as satisfactory (Figure 2). Both nursing care (9; 8–10) and care provided by doctors (8; 7–9) were highly appreciated, as well as the communication, information and support by the medical personnel (8; 8–9, two relatives (4%) unsatisfactory). Thirty-one (53%) relatives reported that the end-of-life phase was neither too long nor too short (score of 4–6). The median length of the end-of-life phase in these patients was 14 h (IQR 4–31). Five (9%) relatives found the dying phase too short (score of 0–2, median length end-of-life phase 10 h; IQR 4–33), and 16 (27%) found this too long (score of 8–10, median length end-of-life phase 32 h; IQR 14–101). The opinion of relatives concerning the duration of the end-of-life phase correlated with the actual length of this phase in hours (r = 0.363; *p* = 0.005).

Results for the quality rating components of the QODD-ICU items are shown in Table 2. The items that were most frequently scored as unsatisfactory by relatives were questions about whether or not the patient ‘was able to feed him/herself’ (69% unsatisfactory), ‘said goodbye to loved ones’ (51% unsatisfactory), ‘appeared to have control over the situation’ (47% unsatisfactory), ‘appeared to keep dignity and self-respect’ (41% unsatisfactory) or ‘appeared to breath comfortably’ (38% unsatisfactory) (Table 2). Results for the frequency components of the QODD items are shown in Supplementary Material Table 1 in the supplemental material. All relatives reported that the patient was accompanied by family most to all of the time and 55 (93%) relatives reported physical contact or hugging the patient. The often acute and unexpected occurrence of stroke was also reflected in the answers of the relatives: only 10 (17%) reported that the patient had had the chance to say goodbye to loved ones. Finally, relatives’ experiences with the project were positive: both the interview (8; 8–8) and the condolence letter (8; 7–9) were appreciated. An overview of relatives’ scores for the quality rating and frequency components of all QODD items is shown in Tables 2 and 3 in the Supplementary Material.

**Table 2.** Summary score and scores on the quality rating component of QODD items.

	Relatives (*n* = 59)			Nurses (*n* = 59)			*p*-value
	*N*	Median (IQR)	Score 0–5 (%)*	*N*	Median (IQR)	Score 0–5 (%)*	
Overall summary score	59	8 [6,8]	9 (15)	56	7 [6,8]	10 (18)	0.18
QODD items							
Appeared to have pain under control	56	8 [6,9]	10 (18)	55	7 [7,8]	6 (11)	0.56
Appeared to have control over the situation	53	6 [3,7]	25 (47)	48	7 [5,8]	15 (31)	<0.01
Was able to feed him/herself	35	5 [5,6]	24 (69)	43	7 [5,8]	11 (26)	<0.01
Appeared to breath comfortably	56	6 [4,8]	21 (38)	55	6 [4,8]	21 (38)	0.35
Appeared to be short of breath	57	6 [4,8]	18 (32)	54	6 [5,8]	18 (33)	0.74
Medication appeared to relieve symptoms of discomfort	44	8 [6,8]	6 (14)	48	8 [7,8]	9 (19)	0.89
Was unafraid of dying	45	8 [6,9]	11 (24)	44	8 [7,9]	7 (16)	0.53
Appeared to keep dignity and self-respect	32	7 [4,8]	13 (41)	41	7 [5,8]	12 (29)	0.85
Spent time with family/friends	59	9 [8,10]	1 (2)	55	9 [8,10]	8 (15)	0.06
Spent time alone	58	9 [8,10]	3 (5)	54	9 [6,10]	9 (17)	0.16
Was touched/hugged by loved ones	57	9 [8,10]	2 (4)	50	8 [7,9]	4 (8)	0.16
Said goodbye to loved ones	53	5 [2,7]	27 (51)	39	5 [3,6]	28 (72)	0.94
Had visit(s) from spiritual advisor	31	8 [6,9]	3 (10)	33	7 [5,8]	14 (42)	0.15

QODD: quality of dying and death; *N:* number of valid responses; IQR: interquartile range.

*Score 0–5 indicates proportion of respondents that scored the item as unsatisfactory. *p*-values are for differences between scores given by relatives and nurses.

### Experiences of nurses

In general, reports of nurses were very much in line with the reports of relatives (Table 2). The only differences were that nurses scored a more positive experience on the quality rating component of the QODD questions about whether the patient ‘appeared to be in control over the situation’ and ‘was able to feed him/herself’. However, as shown by the frequency component of these QODD items (Supplementary Material Table 1), these were rarely applicable to our patients: 48 (84%) relatives and 47 (80%) nurses reported that the patient was never or sometimes (frequency score 0–2) in control of the situation, and 45 (88%) relatives and 56 (95%) nurses reported that the patient was never or sometimes able to feed himself or herself.

### Symptoms of discomfort

The most frequently reported signs of discomfort based on the frequency component of the QODD items were related to breathing: 27 (46%) relatives and 19 nurses (36%) reported that breathing was ‘never or sometimes’ easy. However, 37 (65%) relatives and 41 (71%) nurses reported that symptoms of discomfort were resolved by medication most or all of the time. Pain was not frequently reported: 44 (76%) relatives and 52 (89%) nurses reported that it was under control most or all of the time.

No clinical characteristics were associated with the overall experience of quality of dying in multivariate analysis, but there was a trend towards a more negative experience associated with the use of benzodiazepines (Beta −1.31; *p* = 0.07; Supplementary Material Table 4). In addition, negative experiences with regard to breathing were associated with the use of morphine after adjustment for age and length of the end-of-life phase (Beta −0.27; *p* = 0.05; Supplementary Material Table 5).

## Discussion

In this single-centre evaluation of patients dying on the stroke unit, we found that the majority of relatives were satisfied with the quality of dying and the quality of care provided by nurses and doctors. The most important negative experiences were related to feeding problems, breathing difficulties, not retaining control and sense of dignity and not being able to say goodbye to loved ones. Pain was not frequently reported as a sign of discomfort and relatives were satisfied with the alleviation of symptoms by palliative medication. Experiences of the nurses correlated well with those of the relatives.

Few previous studies have interviewed relatives with an after-death questionnaire to systematically evaluate quality of dying in the stroke unit. To our knowledge, none have used the QODD-ICU to identify frequent symptoms of dying stroke patients and aspects of the dying process itself, and our study is the first to compare experiences of relatives and nurses. A study from the United Kingdom used the Views of Informal Carers Evaluation of Services postal questionnaire in relatives of stroke patients who died in an institutional setting and reported that individualized end-of-life care increased satisfaction.^12^ This study was different from ours because it included also patients who died on hospital wards other than the stroke unit and in nursing homes and used a self-administered questionnaire that had a stronger focus on relatives’ satisfaction with the services provided by healthcare professionals. In line with our findings, a Canadian study that used the After–Death Bereaved Family Member Interview in family members of patients in neurology or neurosurgery services reported overall high levels of satisfaction with palliation and more specifically with treatment of pain and dyspnoea.^13^ However, conclusions were hampered by the small sample size of 15 patients. By contrast, two retrospective chart reviews on dying stroke patients referred to palliative care consultants did identify pain as an important sign of discomfort,^7,14^ which might be explained by the fact that these studies used descriptions of signs of discomfort from the patient charts instead of relatives’ reports. The proportion of patients treated with opioids was comparable to that in our study (around 70%), with 81% of patients reported free of pain in the last 48 h before death in one of the studies.^7^ This may explain the satisfaction with the alleviation of symptoms by palliative medication observed in our study.

Breathing difficulties are among the most frequent reported signs of discomfort in the last hours or days before death at the stroke unit.^7,14^ In our cohort, morphine was used to relieve dyspnoea in 74% of the patients. This is comparable to the use of morphine in 80% of patients who died on a stroke unit in Germany,^15^ but less than in one Canadian study (93.6%).^16^ Our finding that the use of morphine was negatively associated with an experience of discomfort caused by breathing difficulties is probably a matter of confounding by indication. Also, the assessment of breathing difficulties by family members during the whole end-of-life phase might be influenced by periods with changes in the respiratory pattern associated with the dying process before opioids were started. Opioids will probably have alleviated breathing difficulties in most instances since 65% of the interviewed relatives in our study reported that signs of discomfort were successfully resolved by medication most or all of the time. Unfortunately, the effect of non-pharmacological treatments used on our stroke unit, such as repositioning of the patients and suctioning to prevent pooling of saliva in the posterior oropharynx, could not be analysed as they were not systematically documented in the hospital charts.

Assessments of quality of dying by nurses correlated well with those of relatives, suggesting that nurses are a reliable surrogate for relatives of stroke patients in future studies. When analysing the quality component of the individual QODD items, we did, however, find a more positive experience of the nurses on items about the patient ‘feeding him/herself’ and ‘appearing in control over the situation’. As indicated by the frequency component of these items (Supplementary Material Table 1), both are rarely applicable to a dying stroke patient, which can be considered common knowledge to an experienced nurse. However, these aspects may be worrisome to family members, particularly with respect to fluid intake. This is supported by our finding that the item about feeding was amongst the most frequent unsatisfactory experiences of family members. In line with this, American Heart Association guidelines state that it is extremely important to counsel families on what to expect in terms of changing and signs and symptoms, including decreased food and fluid intake and decreased levels of consciousness and agitation.^1^

Several limitations to our analysis should be considered. Firstly, results of our single-centre analysis might be heavily influenced by local protocols and caregivers and cannot be directly generalized to stroke units elsewhere. Secondly, the QODD-ICU has not been validated in acute stroke patients and several of its items were not be applicable to dying stroke patients. Thirdly, nurses completed the questionnaire one week after death to prevent recall bias, whereas relatives completed the questionnaire at 6 weeks to respect their grief. Fourthly, the experiences of relatives may not precisely reflect those of the dying patient him- or herself and perspectives may differ between family members depending on their relationship with the deceased.^8^ Recall of proxies is prone to bias and bereaved informants’ emotions during the dying process may impact their views.^17^ However, evidence from a comprehensive review of the literature of studies comparing patient and proxy views suggested that proxies can reliably report on the quality of services and symptoms, especially on the ones that are more observable in nature.^18^ Fifthly, we could not analyse the impact of advance directives on the experiences of family members during the dying process, as this information is not systematically recorded in our charts. In a recent study from our centre, just one of 49 patients with severe stroke had a written advance directive at admission,^19^ supporting our experience that these are infrequent among stroke patients in the Netherlands. The impact of advance directives, if any, will therefore have been small. Finally, there is a potential for selection bias because just over half of the relatives agreed to be interviewed. Although we found no differences in baseline characteristics and no difference in quality of dying rated by nurses between patients with participating and those with non-participating relatives, we cannot rule out the possibility that relatives who were unsatisfied with the provided end-of-life may been more prone to decline the interview.

## Conclusion

Bereaved family members were satisfied with the quality of dying of patients on our stroke unit, including pain and symptom control and the role of healthcare professionals. Negative experiences during the dying phase were mainly related to feeding problems, breathing difficulties, not retaining sense of dignity and not being able to say goodbye to loved ones. Our results suggest that nurses can reliably assess the experiences of the family members of dying patients and could be used when evaluating end-of-life care for acute stroke patients in future research.

## Supplemental Material

sj-pdf-1-eso-10.1177_23969873211041843 – Supplemental Material for Quality of dying after acute stroke Click here for additional data file. Supplemental Material, sj-pdf-1-eso-10.1177_23969873211041843 for Quality of dying after acute stroke by Hendrik Reinink, Marjolein Geurts, Constance Melis-Riemens, Annemarie D Hollander, L Jaap Kappelle and H Bart van der Worp in European Stroke Journal
