# Supplementary material for: Quality of dying after acute stroke
Source: Eur Stroke J. 2021 Sep 5;6(3):268–75. doi: 10.1177/23969873211041843 (PMC8564161; doi:10.1177/23969873211041843)
Supplement: sj-pdf-1-eso-10.1177_23969873211041843 – Supplemental Material for Quality of dying after acute stroke [file sj-pdf-1-eso-10.1177_23969873211041843.pdf]

SUPPLEMENTAL MATERIAL

Quality of dying after acute stroke

Hendrik Reinink, MD, MSc[1]; Marjolein Geurts, MD, PhD[1,2]; Constance Melis-Riemens[1]; Annemarie D. Hollander[1]; L Jaap Kappelle, MD, PhD[1]; H Bart van der Worp, MD, PhD[1]

[1] Department of Neurology and Neurosurgery, University Medical Center, Utrecht University, Utrecht, The Netherlands

[2] Erasmus Medical Center, University Medical Center Rotterdam, The Netherlands

Table of Contents

Supplemental Methods

Questionnaire for family members p.3
Questionnaire for nurses p.9

Supplemental Tables

Table I–Scores on the frequency component of QODD items p.15
Table II - Distribution of relatives' scores on the quality rating component of QODD items p.16
Table III - Distribution of relatives' scores on the frequency component of QODD items p.17
Table IV - Relationship between clinical characteristics and quality of dying experience p.18
Table V - Relationship between clinical characteristics and experience of shortness of breath p.19

Questionnaire for Family Members

A. General questions

1. Overall, how would you rate the quality of dying of __________?

Terrible experience 0 1 2 3 4 5 6 7 8 9 10 Almost perfect

---

2. What did you think about the length of the dying process?

Much too short 0 1 2 3 4 5 6 7 8 9 10 Much too long

---

3. What was the most unpleasant aspect of the dying process?

---

4. Was there an aspect that you liked? If yes, what?

---

5. How would you rate the care that __________ received in his/her final days from the nurses?

Worst possible care 0 1 2 3 4 5 6 7 8 9 10 Best possible care

---

6. How would you rate the care that __________ received in his/her final days from the doctors?

Worst possible care 0 1 2 3 4 5 6 7 8 9 10 Best possible care

---

7. a. How would you rate the communication, information and support to you by the medical personnel?

Worst possible 0 1 2 3 4 5 6 7 8 9 10 Best Possible

b. Which aspects did you like?

c. Which aspects could be improved?

---

8. a. Did you receive the letter of condolence?

1	2	8
Yes	No	Don't Know

b. How did you experience the letter of condolence?

Terrible experience 0 1 2 3 4 5 6 7 8 9 10 Almost Perfect

c. Please state the reason:

B. Frequency questions

9. a. How often did _____________ appear to have her/his pain under control?

0	1	2	3	4	5	8	9
None of the time	A little bit of the time	Some of the time	A good bit of the time	Most of the time	All of the time	Don't know	No response

b. How would you rate this aspect of _____________ 's dying experience?

Terrible experience 0 1 2 3 4 5 6 7 8 9 10 Almost Perfect

10. a. How often did _____________ appear to have control over what was going on around her/him?

0	1	2	3	4	5	8	9
None of the time	A little bit of the time	Some of the time	A good bit of the time	Most of the time	All of the time	Don't know	No response

b. How would you rate this aspect of _____________ 's dying experience?

Terrible experience 0 1 2 3 4 5 6 7 8 9 10 Almost Perfect

11. How often was _____________ able to feed her/himself?

0	1	2	3	4	5	8	9
None of the time	A little bit of the time	Some of the time	A good bit of the time	Most of the time	All of the time	Don't know	No response

b. How would you rate this aspect of _____________ 's dying experience?

Terrible experience 0 1 2 3 4 5 6 7 8 9 10 Almost Perfect

---

12. a. How often did _____________ breathe comfortably?

0	1	2	3	4	5	8	9
None of the time	A little bit of the time	Some of the time	A good bit of the time	Most of the time	All of the time	Don't know	No response

b. How would you rate this aspect of _____________ 's dying experience?

Terrible experience 0 1 2 3 4 5 6 7 8 9 10 Almost Perfect

---

13. a. How often did _____________ seem short of breath?

0	1	2	3	4	5	8	9
None of the time	A little bit of the time	Some of the time	A good bit of the time	Most of the time	All of the time	Don't know	No response

b. How would you rate this aspect of _____________ 's dying experience?

Terrible experience 0 1 2 3 4 5 6 7 8 9 10 Almost Perfect

---

14. a. How often did medication resolve symptoms of discomfort?

0	1	2	3	4	5	8	9
None of the time	A little bit of the time	Some of the time	A good bit of the time	Most of the time	All of the time	Don't know	No response

b. How would you rate this aspect of _____________ 's dying experience?

Terrible experience 0 1 2 3 4 5 6 7 8 9 10 Almost Perfect

---

15. How often did _____________ appear to be unafraid of dying?

0	1	2	3	4	5	8	9
None of the time	A little bit of the time	Some of the time	A good bit of the time	Most of the time	All of the time	Don't know	No response

b. How would you rate this aspect of _____________ 's dying experience?

Terrible experience 0 1 2 3 4 5 6 7 8 9 10 Almost Perfect

---

16. How often did _____________ appear to feel at peace with dying?

0	1	2	3	4	5	8	9
None of the time	A little bit of the time	Some of the time	A good bit of the time	Most of the time	All of the time	Don't know	No response

b. How would you rate this aspect of _____________ 's dying experience?

Terrible experience 0 1 2 3 4 5 6 7 8 9 10 Almost Perfect

---

17. a. How often did _____________ laugh and smile?

0	1	2	3	4	5	8	9
None of the time	A little bit of the time	Some of the time	A good bit of the time	Most of the time	All of the time	Don't know	No response

b. How would you rate this aspect of _____________ 's dying experience?

Terrible experience 0 1 2 3 4 5 6 7 8 9 10 Almost Perfect

---

18. How often did _____________ appear to keep her/his dignity and self-respect?

0	1	2	3	4	5	8	9
None of the time	A little bit of the time	Some of the time	A good bit of the time	Most of the time	All of the time	Don't know	No response

b. How would you rate this aspect of _____________ 's dying experience?

Terrible experience 0 1 2 3 4 5 6 7 8 9 10 Almost Perfect

---

19. a. How often did_____________ spend time with other family and friends?

0	1	2	3	4	5	8	9
None of the time	A little bit of the time	Some of the time	A good bit of the time	Most of the time	All of the time	Don't know	No response

b. How would you rate this aspect of _____________ 's dying experience?

Terrible experience 0 1 2 3 4 5 6 7 8 9 10 Almost Perfect

---

20. a. How often did _____________ spend time alone?

0	1	2	3	4	5	8	9
None of the time	A little bit of the time	Some of the time	A good bit of the time	Most of the time	All of the time	Don't know	No response

b. How would you rate this aspect of _____________ 's dying experience?

Terrible experience 0 1 2 3 4 5 6 7 8 9 10 Almost Perfect

C. Questions about events

21. a. Was _____________ touched or hugged by her/his loved ones?

1	2	8
Yes	No	Don't Know

b. How would you rate this aspect of _____________ 's dying experience?

Terrible experience 0 1 2 3 4 5 6 7 8 9 10 Almost Perfect

---

22 a. Did _____________ say goodbye to loved ones?

1	2	8
Yes	No	Don't Know

b. How would you rate this aspect of _____________ 's dying experience?

Terrible experience 0 1 2 3 4 5 6 7 8 9 10 Almost Perfect

---

23. a. Did _____________ clear up any bad feelings with others?

1	2	8
Yes	No	Don't Know

b. How would you rate this aspect of _____________ 's dying experience?

Terrible experience 0 1 2 3 4 5 6 7 8 9 10 Almost Perfect

---

24. a. Did _____________ have one or more visits from a religious or spiritual advisor?

1	2	8
Yes	No	Don’t Know

b. How would you rate this aspect of _____________ 's dying experience?

Terrible experience 0 1 2 3 4 5 6 7 8 9 10 Almost Perfect

---

25. a. Did _____________ have a spiritual service or ceremony before his/her death?

1	2	8
Yes	No	Don’t Know

b. How would you rate this aspect of _____________ 's dying experience?

Terrible experience 0 1 2 3 4 5 6 7 8 9 10 Almost Perfect

---

26. a. Did _____________ have her/his funeral arrangements in order prior to death or discuss his/her preferences?

1	2	8
Yes	No	Don’t Know

b. How would you rate this aspect of _____________ 's dying experience?

Terrible experience 0 1 2 3 4 5 6 7 8 9 10 Almost Perfect

---

27. Is there anything else you would like to add?

---

28. How did you experience this interview?

Terrible experience 0 1 2 3 4 5 6 7 8 9 10 Almost Perfect

Questionnaire for nurses

1 a. How often did _____________ appear to have her/his pain under control?

0	1	2	3	4	5	8	9
None of the time	A little bit of the time	Some of the time	A good bit of the time	Most of the time	All of the time	Don’t know	No response

b. How would you rate this aspect of _____________ 's dying experience?

Terrible experience 0 1 2 3 4 5 6 7 8 9 10 Almost Perfect

---

2. **a. How often did _____________ appear to have control over what was going on around her/him?**

0	1	2	3	4	5	8	9
None of the time	A little bit of the time	Some of the time	A good bit of the time	Most of the time	All of the time	Don’t know	No response

b. How would you rate this aspect of _____________ 's dying experience?

Terrible experience 0 1 2 3 4 5 6 7 8 9 10 Almost Perfect

---

3. **a. How often was _____________ able to feed her/himself?**

0	1	2	3	4	5	8	9
None of the time	A little bit of the time	Some of the time	A good bit of the time	Most of the time	All of the time	Don’t know	No response

b. How would you rate this aspect of _____________ 's dying experience?

Terrible experience 0 1 2 3 4 5 6 7 8 9 10 Almost Perfect

---

4. a. How often did _____________ breathe comfortably?

0	1	2	3	4	5	8	9
None of the time	A little bit of the time	Some of the time	A good bit of the time	Most of the time	All of the time	Don’t know	No response

b. How would you rate this aspect of _____________ 's dying experience?

Terrible experience 0 1 2 3 4 5 6 7 8 9 10 Almost Perfect

---

5. a. How often did _____________ seem short of breath?

0	1	2	3	4	5	8	9
None of the time	A little bit of the time	Some of the time	A good bit of the time	Most of the time	All of the time	Don’t know	No response

b. How would you rate this aspect of _____________ 's dying experience?

---

6. a. How often did medication resolve symptoms of discomfort?

0	1	2	3	4	5	8	9
None of the time	A little bit of the time	Some of the time	A good bit of the time	Most of the time	All of the time	Don’t know	No response

b. How would you rate this aspect of _____________ 's dying experience?

Terrible experience 0 1 2 3 4 5 6 7 8 9 10 Almost Perfect

---

7. a. How often did _____________ appear to feel at peace with dying?

0	1	2	3	4	5	8	9
None of the time	A little bit of the time	Some of the time	A good bit of the time	Most of the time	All of the time	Don’t know	No response

b. How would you rate this aspect of _____________ 's dying experience?

Terrible experience 0 1 2 3 4 5 6 7 8 9 10 Almost Perfect

---

8. a. How often did _____________ appear to be unafraid of dying?

0	1	2	3	4	5	8	9
None of the time	A little bit of the time	Some of the time	A good bit of the time	Most of the time	All of the time	Don’t know	No response

b. How would you rate this aspect of _____________ 's dying experience?

Terrible experience 0 1 2 3 4 5 6 7 8 9 10 Almost Perfect

---

9. a. How often did _____________ laugh and smile?

0	1	2	3	4	5	8	9
None of the time	A little bit of the time	Some of the time	A good bit of the time	Most of the time	All of the time	Don't know	No response

b. How would you rate this aspect of _____________ 's dying experience?

Terrible experience 0 1 2 3 4 5 6 7 8 9 10 Almost Perfect

---

10. a. How often did _____________ appear to keep her/his dignity and self-respect?

0	1	2	3	4	5	8	9
None of the time	A little bit of the time	Some of the time	A good bit of the time	Most of the time	All of the time	Don't know	No response

b. How would you rate this aspect of _____________ 's dying experience?

Terrible experience 0 1 2 3 4 5 6 7 8 9 10 Almost Perfect

---

11. a. How often did_____________ spend time with other family and friends?

0	1	2	3	4	5	8	9
None of the time	A little bit of the time	Some of the time	A good bit of the time	Most of the time	All of the time	Don't know	No response

b. How would you rate this aspect of _____________ 's dying experience?

Terrible experience 0 1 2 3 4 5 6 7 8 9 10 Almost Perfect

---

12. a. How often did _____________ spend time alone?

0	1	2	3	4	5	8	9
None of the time	A little bit of the time	Some of the time	A good bit of the time	Most of the time	All of the time	Don't know	No response

b. How would you rate this aspect of _____________ 's dying experience?

Terrible experience 0 1 2 3 4 5 6 7 8 9 10 Almost Perfect

---

13. a. Was _____________ touched or hugged by her/his loved ones?

1	2	8
Yes	No	Don't Know

b. How would you rate this aspect of _____________ 's dying experience?

Terrible experience 0 1 2 3 4 5 6 7 8 9 10 Almost Perfect

---

14. a. Did _____________ say goodbye to loved ones?

1	2	8
Yes	No	Don't Know

b. How would you rate this aspect of _____________ 's dying experience?

Terrible experience 0 1 2 3 4 5 6 7 8 9 10 Almost Perfect

---

15. a. Did _____________ have one or more visits from a religious or spiritual advisor?

1	2	8
Yes	No	Don't Know

b. How would you rate this aspect of _____________ 's dying experience?

Terrible experience 0 1 2 3 4 5 6 7 8 9 10 Almost Perfect

---

16. a. Did _____________ have a spiritual service or ceremony before his/her death?

1	2	8
Yes	No	Don't Know

b. How would you rate this aspect of _____________ 's dying experience?

Terrible experience 0 1 2 3 4 5 6 7 8 9 10 Almost Perfect

---

17. What did you think about the length of the dying process?

Much too short 0 1 2 3 4 5 6 7 8 9 10 Much too long

---

18. What was the most unpleasant aspect of the dying process?

---

19. Was there an aspect that you liked? If yes, what?

---

20. Overall, how would you rate the quality of dying of __________?

Terrible experience 0 1 2 3 4 5 6 7 8 9 10 Almost perfect

Supplemental Tables

	Relatives (n = 59)					Nurses (n = 59)				
	N	0-2 (%)	3-5 (%)	Don't know	No response	N	0-2 (%)	3-5 (%)	Don't know	No response
**Frequency QODD items**										
Appeared to have pain under control	58	5 (9)	44 (76)	9 (15)	0 (0)	59	5 (8)	52 (89)	2 (3)	0 (0)
Appeared to have control over the situation	57	48 (84)	2 (4)	6 (10)	1 (2)	59	47 (80)	5 (8)	5 (8)	2 (3)
Was able to feed him/herself	51	45 (88)	0 (0)	3 (5)	3 (5)	59	56 (95)	0 (0)	1 (2)	2 (3)
Appeared to breath comfortably	59	27 (46)	31 (53)	1 (2)	0 (0)	59	22 (37)	36 (61)	1 (2)	0 (0)
Appeared to be short of breath	59	47 (80)	10 (17)	2 (3)	0 (0)	59	47 (80)	10 (17)	2 (3)	0 (0)
Medication appeared to relieve symptoms of discomfort	57	6 (11)	37 (65)	14 (24)	0 (0)	58	6 (10)	41 (71)	10 (17)	1 (2)
Was unafraid of dying	59	27 (46)	16 (27)	15 (25)	1 (2)	58	18 (31)	23 (40)	15 (26)	2 (3)
Appeared to keep his/her dignity and self-respect	59	7 (12)	17 (29)	32 (54)	3 (5)	59	9 (15)	26 (44)	23 (39)	1 (2)
Spent time with family/friends	59	0 (0)	59 (100)	0 (0)	0 (0)	56	6 (11)	50 (89)	0 (0)	0 (0)
Spent time alone	59	53 (90)	5 (8)	1 (2)	0 (0)	59	50 (85)	7 (12)	0 (0)	2 (3)
**Yes/no QODD items**	**N**	**Yes**	**No**	**Don't know**		**N**	**Yes**	**No**	**Don't know**	
Was touched or hugged by loved ones	59	55 (93)	3 (5)	1 (2)		59	50 (85)	2 (3)	7 (12)	
Said goodbye to loved ones	58	10 (17)	44 (76)	4 (7)		59	7 (12)	34 (58)	18 (31)	
Had visit(s) from spiritual advisor	59	9 (15)	24 (41)	26 (44)		59	2 (3)	44 (75)	13 (22)	

Table I – scores on the frequency component of QODD items. N is the number of valid responses. The upper panel shows the proportion of relatives or nurses that scored the frequency component as occurring "never or sometimes (score 0-2)", "most or all of the time" (score 3-5), "don't know" (score 8) or "no response" (score 9). The lower panel shows the answers of relatives and nurses to dichotomous questions that asked whether situations occurred during the end of life phase ("yes") or did not occur ("no").

	Relatives' score (%)											
	N	0 (%)	1 (%)	2 (%)	3 (%)	4 (%)	5 (%)	6 (%)	7 (%)	8 (%)	9 (%)	10 (%)
Overall summary score	59	3 (5.1)	0 (0)	1 (1.7)	1 (1.7)	2 (3.4)	2 (3.4)	6 (10.2)	11 (18.6)	19 (32.2)	9 (15.3)	5 (8.5)
Appeared to have pain under control	56	1 (1.8)	1 (1.8)	2 (3.6)	1 (1.8)	1 (1.8)	4 (7.1)	4 (7.1)	8 (14.3)	17 (30.4)	5 (8.9)	12 (21.4)
Appeared to have control over the situation	53	8 (15.1)	3 (5.7)	2 (3.8)	3 (5.7)	4 (7.5)	5 (9.4)	5 (9.4)	13 (24.5)	8 (15.1)	2 (3.8)	0 (0)
Was able to feed him/herself	35	4 (11.4)	0 (0)	0 (0)	2 (5.7)	1 (2.9)	17 (48.6)	5 (14.3)	3 (8.6)	1 (2.9)	1 (2.9)	1 (2.9)
Appeared to breath comfortably	56	5 (8.9)	2 (3.6)	3 (5.4)	3 (5.4)	4 (7.1)	4 (7.1)	9 (16.1)	8 (14.3)	11 (19.6)	3 (5.4)	4 (7.1)
Appeared to be short of breath	57	4 (7.0)	0 (0)	3 (5.3)	5 (8.8)	3 (5.3)	3 (5.3)	11 (19.3)	4 (7.0)	13 (22.8)	1 (1.8)	10 (17.5)
Medication appeared to relieve symptoms of discomfort	44	1 (2.3)	0 (0)	0 (0)	1 (2.3)	2 (4.5)	2 (4.5)	5 (11.4)	9 (20.5)	17 (38.6)	4 (9.1)	3 (6.8)
Was unafraid of dying	45	3 (6.7)	0 (0)	1 (2.2)	2 (4.4)	3 (6.7)	2 (4.4)	2 (4.4)	2 (4.4)	15 (33.3)	5 (11.1)	10 (22.2)
Appeared to keep dignity and self-respect	32	1 (3.1)	1 (3.1)	2 (6.3)	3 (9.4)	1 (3.1)	5 (15.6)	2 (6.3)	3 (9.4)	8 (25.0)	1 (3.1)	5 (15.6)
Spent time with family/friends	59	0 (0)	0 (0)	0 (0)	0 (0)	1 (1.7)	0 (0)	1 (1.7)	2 (3.4)	22 (37.3)	7 (11.9)	26 (44.1)
Spent time alone	58	0 (0)	0 (0)	1 (1.7)	1 (1.7)	1 (1.7)	0 (0)	3 (5.2)	7 (12.1)	15 (25.9)	9 (15.5)	21 (36.2)
Was touched/hugged by loved ones	57	0 (0)	0 (0)	1 (1.8)	0 (0)	1 (1.8)	0 (0)	3 (5.3)	2 (3.5)	21 (36.8)	10 (17.5)	19 (33.3)
Said goodbye to loved ones	53	8 (15.1)	5 (9.4)	2 (3.8)	6 (11.3)	2 (3.8)	4 (7.5)	6 (11.3)	9 (17.0)	5 (9.4)	3 (5.7)	3 (5.7)
Had visit(s) from spiritual advisor	31	0 (0)	0 (0)	0 (0)	0 (0)	1 (3.2)	2 (6.5)	6 (19.4)	6 (19.4)	6 (19.4)	3 (9.7)	7 (22.6)

Table II - Distribution of all relatives' scores on the quality rating component of QODD items. *QODD indicates quality of dying and death. N is the number of valid responses per QODD item.*

	Relatives (n = 59)								
	N	0 (%)	1 (%)	2 (%)	3 (%)	4 (%)	5 (%)	Don't know (%)	No response (%)
**Frequency QODD items**									
Appeared to have pain under control	58	1 (1.7)	0 (0)	4 (6.9)	4 (6.9)	10 (17.2)	30 (51.7)	9 (15.5)	0 (0)
Appeared to have control over the situation	57	30 (52.6)	10 (17.5)	8 (14.0)	1 (1.8)	1 (1.8)	0 (0)	6 (10.5)	1 (1.8)
Was able to feed him/herself	51	41 (80.4))	2 (3.9)	2 (3.9)	0 (0)	0 (0)	0 (0)	3 (5.9)	3 (5.9)
Appeared to breath comfortably	59	5 (8.5)	4 (6.8)	18 (30.5)	10 (16.9)	13 (22.0)	8 (13.6)	1 (1.7)	0 (0)
Appeared to be short of breath	59	25 (42.4)	5 (8.5)	17 (28.8)	7 (11.9)	3 (5.1)	0 (0)	2 (3.4)	0 (0)
Medication appeared to relieve symptoms of discomfort	57	0 (0)	3 (5.3)	3 (5.3)	6 (10.5)	15 (26.3)	16 (28.1)	14 (24.6)	0 (0)
Was unafraid of dying	59	20 (33.9)	3 (5.1)	4 (6.8)	1 (1.7)	2 (3.4)	13 (22.0)	15 (25.4)	1 (1.7)
Appeared to keep his/her dignity and self-respect	59	4 (6.8)	2 (3.4)	1 (1.7)	0 (0)	6 (10.2)	11 (18.6)	32 (54.2)	3 (5.1)
Spent time with family/friends	59	0 (0)	0 (0)	0 (0)	8 (13.6)	10 (16.9)	41 (69.5)	0 (0)	0 (0)
Spent time alone	59	35 (59.3)	8 (13.6)	10 (16.9)	4 (6.8)	1 (1.7)	0 (0)	1 (1.7)	0 (0)
	N	Yes (%)	No (%)					Don't know (%)	
**Yes/no QODD items**									
Was touched or hugged by loved ones	59	55 (93.2)	3 (5.1)					1 (1.7)	
Said goodbye to loved ones	58	10 (17.2)	44 (75.9)					4 (6.9)	
Had visit(s) from spiritual advisor	59	9 (15.3)	24 (40.7)					26 (44.1)	

Table III - Distribution of all relatives' scores on the frequency component of QODD items. *QODD indicates quality of dying and death. N is the number of valid responses per QODD item.*

**Variable**	**Univariate (beta)**	**p-value**	**Multivariate (beta)**	**p-value**
Age	0.02	0.52		
Male sex (vs female sex)	0.25	0.69		
SAH (vs ischemic stroke)	0.96	0.24		
ICH (vs ischemic stroke)	0.24	0.70		
Latest recorded GCS before end-of-life phase	-0.15	0.19		
Use of morphine (yes vs no)	-0.24	0.74		
Use of benzodiazepines (yes vs no)	-1.57	0.02	-1.31	0.07
Length of end-of-life phase (hours)	-0.01	0.06	-0.01	0.18
Time to end-of-life phase (hours)	-0.21	0.10		
Location ICU (vs stroke unit)	-0.46	0.56		
Location ER (vs stroke unit)	0.41	0.54		

Table IV – Relationship between clinical characteristics and quality of dying experience. Analyses are performed by univariate and multivariate linear regression models using the relatives score on the single summary score as the independent variable. SAH indicates subarachnoid hemorrhage; ICH, intracerebral hemorrhage; GCS, Glasgow Coma Scale; location, location where end-of-life care was initiated; ICU, intensive care unit; ER, emergency department. Time to end-of-life phase indicates the time between start of hospital treatment and decision to withdraw life-sustaining measures.

**Variable**	**Univariate (beta)**	**p-value**	**Multivariate (beta)**	**p-value**
Age	0.05	0.13		
Male sex (vs female sex)	-1.04	0.19		
SAH (vs ischemic stroke)	0.69	0.50		
ICH (vs ischemic stroke)	-0.41	0.61		
Latest recorded GCS before end-of-life phase	-0.09	0.52		
Use of morphine (yes vs no)	-2.35	0.01	-0.27	0.05
Use of benzodiazepines (yes vs no)	-0.02	0.98		
Length of end-of-life phase (hours)	-0.01	0.08	-0.13	0.34
Time to end-of-life phase (hours)	-0.24	0.07	-0.14	0.30
Location ICU (vs stroke unit)	-0.06	0.95		
Location ER (vs stroke unit)	1.21	0.15		

Table V– Relationship between clinical characteristics and experience of shortness of breath. Analyses are performed by univariate and multivariate linear regression models using the quality component of the QODD item about shortness of breath as the independent variable. SAH indicates subarachnoid hemorrhage; ICH, intracerebral hemorrhage; GCS, Glasgow Coma Scale; location, location where end-of-life care was initiated; ICU, intensive care unit; ER, emergency department. Time to end-of-life phase indicates the time between start of hospital treatment and decision to withdraw life-sustaining measures.
